# Development and validation of a nomogram for predicting morbidity in surgically resected primary retroperitoneal sarcoma

**DOI:** 10.1186/s12893-023-01941-8

**Published:** 2023-02-23

**Authors:** Aobo Zhuang, Yangju Chen, Lijie Ma, Yuan Fang, Hua Yang, Weiqi Lu, Yuhong Zhou, Yong Zhang, Hanxing Tong

**Affiliations:** 1 Department of Neurology, Department of Medical Oncology, Department of General Surgery, Zhongshan Hospital, Fudan University, Shanghai, People’s Republic of China; 2 Xiamen University Research Center of Retroperitoneal Tumor Committee of Oncology Society of Chinese Medical Association, Xiamen University, Xiamen, Fujian China

**Keywords:** Retroperitoneal sarcoma, Surgery, Postoperative morbidity, Nomogram

## Abstract

**Background:**

Surgery is the cornerstone of the treatment for primary retroperitoneal sarcoma (RPS). The purpose of this study was to establish a nomogram predictive model for predicting postoperative morbidity in primary RPS.

**Methods:**

Clinicopathological data of patients who underwent radical resection from 2009 to 2021 were retrospectively analyzed. Risk factor analysis was performed using a logistic regression model, and modeling variables were selected based on Akaike Information Criterion. The nomogram prediction model was built on the basis of a binary logistic regression model and internally validated by calibration curves and concordance index.

**Results:**

A total of 319 patients were enrolled, including 162 males (50.8%). 22.9% (n = 73) were over 65 years of age, and 70.2% (n = 224) had tumors larger than 10 cm. The most common histologic subtypes were well-differentiated liposarcoma (38.2%), dedifferentiated liposarcoma (25.1%) and leiomyosarcoma (7.8%). According to the Clavien–Dindo Classification, 96 (31.1%) and 31 (11.6%) patients had grade I–II complications and grade III–V complications, respectively. Age, tumor burden, location, operative time, number of combined organ resections, weighted resected organ score, estimated blood loss and packed RBC transfusion was used to construct the nomogram, and the concordance index of which was 0.795 (95% CI 0.746–0.844). and the calibration curve indicated a high agreement between predicted and actual rates.

**Conclusions:**

Nomogram, a visual predictive tool that integrates multiple clinicopathological factors, can help physicians screen RPS patients at high risk for postoperative complications and provide a basis for early intervention.

## Background

Soft tissue sarcoma is a rare tumor, accounting for only about 1% of all solid tumors. Of these, 15% were retroperitoneal sarcomas (RPS) [1]. Surgical resection remains the mainstay of treatment for primary RPS [2]. Since RPS is asymptomatic in its early stages, the tumor burden is often huge at the time of diagnosis. Meanwhile, as reported by Gronchi [3] and Bonvalot [4], aggressive surgical strategies may help reduce the local recurrence of the disease. Therefore, an increasing number of high-volume sarcoma centers now advocate multivisceral resection (MVR). So the median number of combined organ resections for RPS in many hospitals may be as high as 4–5 [5]. The larger the surgery and the longer the operative time, the more complications there will be. The incidence of complications within 30 days after surgery has been reported to be more than 20% [6]. And the incidence of serious postoperative complications is as high as about 15%, and the rate of unplanned reoperations exceeds 10% [7]. In contrast, the proportion of unplanned reoperations for routine general surgery was only 3.5% [8].

A number of studies focused on the perioperative safety of RPS, and age, tumor burden, number of combined organ resections, and packed RBC transfusion have been reported as risk factors for postoperative morbidity [6, 7, 9]. However, there is no predictive tool at present that integrates multiple clinicopathological factors to predict postoperative complications.

The nomogram prediction model, which converts complex regression equations into visual graphs for patient assessment, is gradually gaining more and more attention and application in medical research and clinical practice. There are many high-quality nomogram prediction models for primary RPS, but the primary endpoint of these studies is long-term prognosis [3, 10, 11], and there is currently no nomogram prediction model for postoperative complications alone.

Therefore, the purpose of this study was to establish a nomogram prediction model for postoperative complications in patients with primary RPS, thereby providing a more accurate tool that would help clinicians involved in patient care and clinical research.

## Methods

### Patients

All patients with RPS who underwent curative surgery in the Shanghai Public Health Clinical Center, Fudan University, Shanghai, China from August 2009 to December 2021 were included. The inclusion criteria were as follows: (1) primary disease, (2) histologically confirmed sarcoma, (3) tumors originating from retroperitoneum, and (4) complete clinical pathological information and follow-up information. Patients with Ewing sarcoma, rhabdomyosarcoma, desmoid sarcoma, gynecologic sarcoma, or gastrointestinal stromal tumor were excluded. This study was approved by the Ethics Committee of Shanghai Public Health Clinical Center and was conducted in accordance with the Declaration of Helsinki.

### Surgical procedure

All surgeries were performed by the multidisciplinary team for soft tissue sarcoma at Zhongshan Hospital Affiliated to Fudan University. Well differentiated liposarcoma and low-grade dedifferentiated liposarcoma are mainly local recurrences, so we implement a more aggressive surgical strategy (even if the surrounding organs of the tumor are not violated by the naked eye, they will be resected together); for high-grade dedifferentiated liposarcoma, If it is evaluated that there is the invasion of surrounding organs, complete radical resection should be attempted, and combined organ resection should also be performed; leiomyosarcoma often presents as a tumor with clear borders, and if the surrounding organs invade the adjacent units of the tumor, it should be preserved; for pleomorphic undifferentiated sarcoma, malignant peripheral nerve sheath tumor, and solitary fibrous tumor, complete resection with negative margins is enough.

### Clinicopathologic evaluation

We collected the baseline characteristics that might affect the patient's prognosis such as gender, age, tumor burden, and histologic subtypes. Surgery-related indicators such as operative time, intraoperative blood loss, and the number of combined organ resections were also collected. Among them, the physical status of patients before anesthesia was assessed according to the American Society of Anesthesiologists Physical Status (ASA score) [12]. Tumor burden was the sum of the maximum diameters of all tumors in the patient. According to the 2020 World Health Organization pathological classification, the pathological types were classified as follows: (1) well-differentiated liposarcoma; (2) dedifferentiated liposarcoma; (3) leiomyosarcoma; (4) solitary fibroma; and (5) Others [13]. Meanwhile, according to the French Federation of Centers for the Fight against Cancer (FNCLCC) criteria, tumors were graded into I, II, and III [13]. Complete resection was defined as grossly negative margins, including both R0 and R1 resections. In 2017, realizing that the removal of specific organs may mean more complications, the Transatlantic RPS Working Group scored the different organs removed, as follows: Adrenal gland, aortocaval lymph nodes, appendix, gallbladder, inguinal ligament, omentum, psoas fascia, and skin are 0 points; Adnexa and/or uterus, bladder, bone, diaphragm,

distal pancreas, duodenum or duodenojejunal flexure, femoral/sciatic/obturator nerve or lumbar/sacral nerve root, iliac artery and/or aorta, iliac vein and/or IVC, kidney, left colon and/or rectum, liver, lung, parietal muscles, pericardium, posterior vaginal wall, prostate (with or without seminal vesicle), psoas muscle, right colon, small bowl, spleen, stomach, testis and/ or spermatic cord and/or vas deferens, and ureter (complete or partial resection not associated with nephrectomy) is 1 point; pancreaticoduodenectomy is 2 points [7]. According to the above scoring criteria, we scored each patient's organ resection.

Postoperative complications within 30 days were the primary endpoint of this study, and were classified according to the Clavien–Dindo Classification [14] as follows: I, abnormal conditions not requiring medical therapy or surgery, endoscopy, and radiation therapy; II, complications requiring medical therapy other than Level I complications; III, complications requiring surgery, endoscopy, or radiation therapy Complications; IV, life-threatening complications; V, postoperative death. Among them, grade III or above was defined as serious postoperative complications.

### Statistical analyses

Overall survival was defined as the time from surgery to death or loss to follow-up, and we used the Kaplan–Meier method to calculate the overall survival rate. Categorical variables were described as numbers and percentages and compared using Fisher's exact test or Pearson Chi-square. Continuous variables were described by means and standard deviations and compared using an independent sample t-test. Univariate analysis were performed using binary logistic regression, and variables with p < 0.05 were further included in multivariate analysis. Binary logistic regression was also used for Multivariate analyses.

For variable selection, all clinicopathological variables were included and a backward procedure based on the Akaike Information Criteria (AIC) was applied [15]. A nomogram based on a multivariate logistic model was then built. Discrimination was assessed using Harrell C's Concordance Index (c-index). Means and 95% confidence intervals were calculated and plotted for each subgroup. Decision curve analysis (DCA) was used to assess the clinical potential application value of the nomogram.

All tests were two-tailed and P < 0.05 was considered statistically significant. All data were analyzed using SPSS 22.0 (SPSS Inc., Chicago, IL, USA) and R 4.0.4 (R Foundation for Statistical Computing, Vienna, Austria; http://www.r-project.org/).

## Results

### Baseline characteristics

**Fig. 1:**
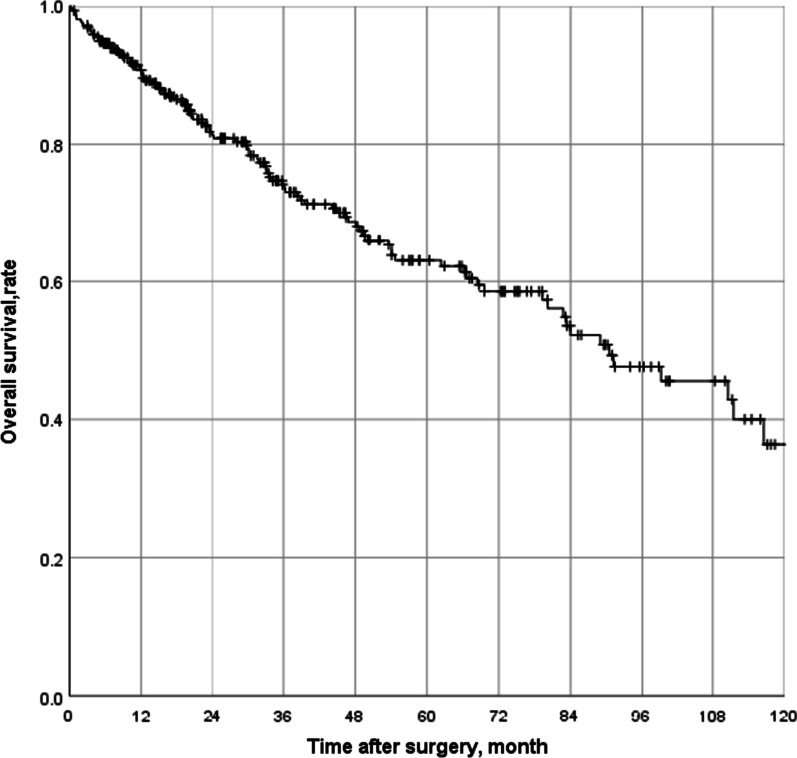
Overall survival in patients with primary retroperitoneal sarcoma

A total of 319 patients were enrolled, and the mean follow-up time for all patients was 45 (range, 1–140) months. 5-year overall survival rate was 63.0 (95%CI, 56.3–69.7) % (Fig. 1). Baseline characteristics of patients and tumors are listed in Table 1. The proportions of men and women were similar (50.8% vs. 49.2%), as were the proportions of symptomatic and asymptomatic patients (43.3% vs. 56.7%). 22.9% of patients were older than 65 years, and 95 patients (29.8%) had a tumor burden greater than 10 cm. Among the histologic subtypes, well-differentiated liposarcoma, dedifferentiated liposarcoma, leiomyosarcoma, and solitary fibroma accounted for 38.2%, 25.1%, 16.3%, and 7.8%, respectively. 31 patients (9.7%) had multifocal disease. 7.8% (n = 25) of patients had received radiotherapy, and 11.0% (n = 35) had received chemotherapy.

**Table 1:** Baseline characteristics of patients and tumors in 319 patients with primary retroperitoneal sarcoma

Characteristics	Number (n = 319)	%
Gender		
Male	162	50.8
Female	157	49.2
Age > 65 years		
Yes	73	22.9
No	246	77.1
ASA score		
1	216	67.7
> 1	103	32.3
Symptoms		
Yes	138	43.3
No	181	56.7
Tumor burden > 10 cm		
Yes	224	70.2
No	95	29.8
Histologic subtypes		
WDLPS	122	38.2
DDLPS	80	25.1
LMS	52	16.3
SFT	25	7.8
Others	40	12.5
UPS	16	5.0
MLPS	14	4.4
MPNST	7	2.2
PLPS	3	0.9
FNCLCC		
Grade 1	113	35.4
Grade 2	104	32.6
Grade 3	91	28.5
Unknown	11	3.4
Location		
Left	160	50.2
Right	159	49.8
Multifocality		
Yes	31	9.7
No	288	90.3
Radiation		
Yes	25	7.8
No	294	92.2
Chemotherapy		
Yes	35	11.0
No	284	89.0

Table 2 presents information related to the surgical procedure. The vast majority of patients underwent open surgery (98.1%) and achieved complete tumor resection (96.9%). 51.1% (n = 163) of patients received more than one combined organ resection, with the most frequently resected organs being the colon (47.3%), kidney (43.6%) and adrenal gland (18.2%). As for the weighted resected organ score, 28.2% (n = 90) of the patients had a score of 0, 25.3% (n = 81) of the patients had a score of 1, and about half of the patients (46.5%) had a score of greater than 1. 90 patients (n = 28.2%) had an operative time more than 4 h, and about half (48.6%) had intraoperative blood loss estimated to be more than 400 ml. 31.3% (n = 100) of patients received packed RBC transfusion. The median postoperative hospital stay for all patients was 18.4 days. The overall complication rate was 42.3%, with 31.1% of grade I–II complications. The serious complication rate was 11.6%, and the most common adverse events were bowel anastomotic leak/fistula (n = 6), sepsis(n = 5), and bleeding/hematoma(n = 4) (Table 3). Six postoperative death, three of them died of bowel anastomotic leak/fistula, one died of sepsis, one died of severe intra-abdominal infection, and one died of myocardial infarction. The 30-, 60-, and 90-day mortality rates were 0.9 (95%CI, 0.3–1.5) %, 2.2 (95%CI, 0.6–3.8)%, and 2.8 (95%CI, 1.0–4.6)%, respectively.

**Table 2:** Surgical characteristics in 319 patients with primary retroperitoneal sarcoma

Characteristics	Number (n = 319)	%
Operation		
Laparoscopic surgery	6	1.9
Open surgery	313	98.1
Complete resection		
Yes	309	96.9
No	10	3.1
Number of combined resections		
0–1	156	48.9
> 1	163	51.1
Resected organs		
Colon	151	47.3
Kidney	139	43.6
Adrenal gland	58	18.2
Small intestine	43	13.5
Spleen	39	12.2
Pancreas	33	10.3
Diaphragm	26	8.2
Abdominal wall	18	5.6
Operative time > 4 h		
Yes	90	28.2
No	229	71.8
Weighted resected organ score		
0	90	28.2
1	81	25.3
2	80	25.0
3	31	9.7
4	21	6.4
5	11	3.4
6	5	1.5
Estimated blood loss > 400 ml		
Yes	155	48.6
No	164	51.4
Packed RBC transfusion		
Yes	100	31.3
No	219	68.7
ICU stay		
Yes	159	49.8
No	160	50.2
Clavien–Dindo Classification		
I–II	96	31.1
III	21	6.6
IV	10	3.1
V	6	1.9
Postoperative hospital stay, days mean (SD)	18.4	13.2

**Table 3:** Type and incidence of all serious complications within 30 days after surgery

Characteristics	Number (n = 319)	%
Bowel anastomotic leak/bowel fistula/gastric fistula	6	1.8
Sepsis	5	1.6
Postoperative bleeding/	4	1.3
Pleural effusion	4	1.3
Abscess	3	0.9
Wound infection/wound healing complications	3	0.9
Abdominal/retroperitoneal collection	3	0.9
Urine leak	2	0.6
Cardiac ischemia/infarct/failure	2	0.6
Others	5	1.6

### Feature selection

To select the best variables for the predictive model, we first performed a risk factor analysis (Table 4). Univariate analysis indicated age > 65 years (p = 0.015), tumor burden > 10 cm (p < 0.001), pathological type (p = 0.046), number of combined organ resections > 1 (p < 0.001), weighted resected organ score (continuous, p < 0.001), operation time > 4 h (p < 0.001), estimated blood loss > 400 ml (p < 0.001) and packed RBC transfusion (p < 0.001) were risk factors for complications. We further included the above risk factors in multivariate analysis. Through multivariate analysis, we found that number of combined organ resections > 1 (HR = 2.864, p = 0.012), weighted resected organ score (HR = 2.041, p < 0.001) and operation time > 4 h (HR = 2.074, p = 0.032) were independent risk factors for complications.

**Table 4:** Univariable and multivariable analyses to determine independent predictors of postoperative complications of primary retroperitoneal sarcoma

Variables	Univariate analysis		Multivariate analysis	
	Hazard ratio (95%CI)	P value	Hazard ratio (95%CI)	P value
Gender male vs. female	1.394 (0.892–2.177)	0.145		
Age > 65 vs ≤ 65 years	1.927 (1.137–3.265)	0.015	1.836 (0.999–3.374)	0.050
ASA score > 1 vs. 1	1.372 (0.855–2.201)	0.190		
Symptoms yes vs. no	1.032 (0.659–1.615)	0.891		
Tumor burden > 10 vs ≤ 10 cm	3.378 (1.961–5.819)	< 0.001	1.786 (0.930–3.430)	0.081
Histologic subtypes		0.046		0.314
DDLPS vs. WDLPS	1.239 (0.704–2.180)		0.698 (0.350–1.394)	
LMS vs. WDLPS	0.393 (0.191–0.808)		0.682 (0.270–1.722)	
SFT vs. WDLPS	0.786 (0.327–1.886)		1.143 (0.454–2.876)	
Others vs. WDLPS	0.707 (0.340–1.471)		2.012 (0.683–5.931)	
FNCLCC		0.636		
Grade 2 vs. Grade 1	1.198 (0.698–2.057)			
Grade 3 vs. Grade 1	1.239 (0.709–2.167)			
Unknown vs. Grade 1	0.567 (0.143–2.251)			
Location left vs. right	1.092 (0.700–1.703)	0.698		
Multifocality yes vs. no	1.515 (0.721–3.183)	0.273		
Radiation yes vs. no	0.750 (0.321–1.752)	0.506		
Chemotherapy yes vs. no	1.025 (0.504–2.084)	0.946		
Operation laparoscopic vs. open	0.267 (0.031–2.314)	0.231		
Complete Resection yes vs. no	1.377 (0.391–4.854)	0.619		
Number of combined resections > 1 vs. 0–1	2.576 (1.628–4.074)	< 0.001	2.864 (1.261–6.507)	0.012
Weighted resected organ score (continuous)	1.894 (1.563–2.296)	< 0.001	2.041 (1.454–2.865)	< 0.001
Operative time > 4 vs. ≤ 4 h	5.088 (2.994–8.646)	< 0.001	2.074 (1.064–4.043)	0.032
Estimated blood loss > 400 vs. ≤ 400 ml	4.109 (2.560–6.595)	< 0.001	1.594 (0.807–3.150)	0.179
Packed RBC transfusion yes vs. no	1.315 (1.163–1.487)	< 0.001	1.726 (0.888–3.357)	0.108
ICU Stay yes vs. no	2.669 (1.687–4.221)	< 0.001	1.259 (0.661–2.396)	0.484

A backward stepwise selection was then performed with all clinicopathological factors using a likelihood ratio test with AIC as the stopping rule. Age, tumor burden, location, operative time, number of combined organ resections, weighted resected organ score, estimated blood loss and packed RBC transfusion was selected. In addition to location, all other variables have statistical differences in univariate analysis.

### Nomogram development and validation

**Fig. 2:**
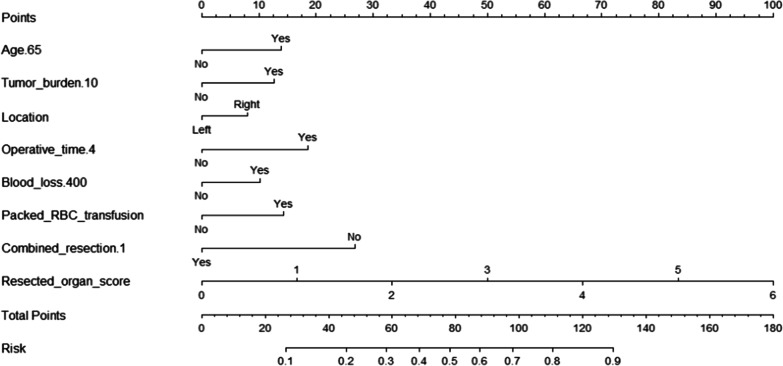
Nomogram for postoperative complications in patients with primary retroperitoneal sarcoma

We developed a nomogram prediction model based on binomial logistic regression with the selected variables above (Fig. 2). In this nomogram prediction model, each clinicopathological risk factor corresponded to a score. The total score could be obtained by summing the scores of the above seven indicators, and the probability of postoperative complications for patients could be inferred from the total score.

**Fig. 3:**
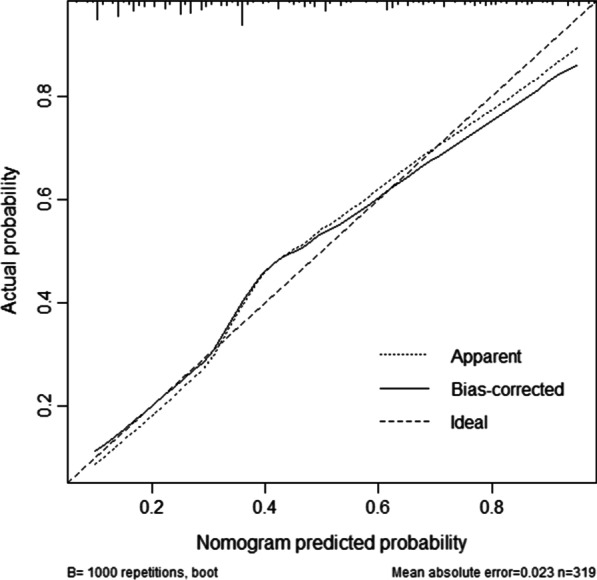
Calibration plots for internal validation of postoperative complication nomogram

The calibration curve in Fig. 3 shows that the predicted postoperative complication probability is highly consistent with the actual probability. And the Harrell C-index for the nomogram was 0.795 (95% CI 0.746–0.844).

**Fig. 4:**
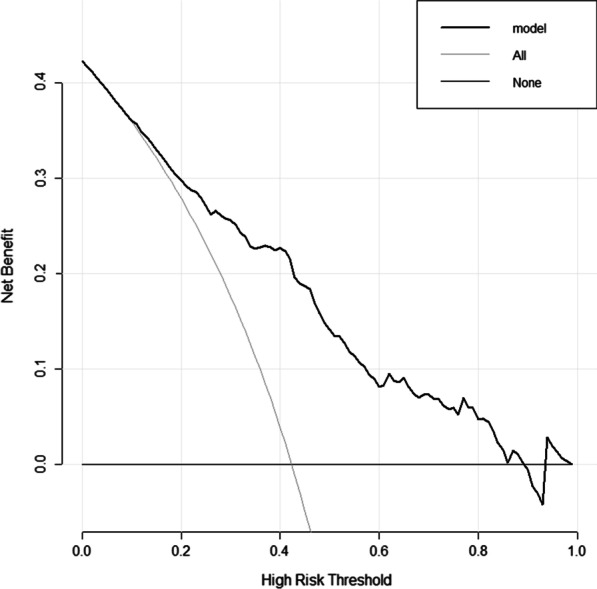
Decision curve analysis for the morbidity predicting model

As illustrated in Fig. 4, the model demonstrated a significant positive net benefit from the risk of postoperative complication, indicating its great clinical practical value in predicting postoperative complications.

## Discussion

Surgery remains the most fundamental treatment for primary RPS [2], which often requires combined multiple organ resection. However, the occurrence of postoperative complications not only prolongs the postoperative hospital stay but also increases the healthcare burden [16]. Therefore, it is particularly important to be able to identify patients at high risk of complications and to intervene early. Consistent with previous studies, we found that age, tumor burden, and operation time were risk factors for postoperative complications in RPS patients. Further, we developed the first nomogram prediction model with selected clinicopathological factors to accurately predict the probability of postoperative complications in patients with primary RPS.

Age has long been a risk factor for postoperative complications. A study by the Transatlantic RPS Working Group on the perioperative safety of 1007 patients with primary RPS also found that age was an independent risk factor for serious postoperative complications [5]. Sourrouille et al. also noted that individuals over 70 years of age undergoing RPS had a mortality rate of 8% and a serious postoperative complication rate of 32%. However, it is noteworthy that the 5-year recurrence-free survival rate of elderly patients can also reach 52% [17]. Therefore, age is not a contraindication to RPS surgery. Some insights may be drawn from the nomogram prediction model in this study. If a patient older than 65 years with a tumor burden less than 10 cm, when the operative time is greater than 4 h, estimated blood loss > 400 ml, and packed RBC transfusion, the patient’s total score was 252 points, meaning the likelihood of postoperative complications was greater than 70%. On the contrary, if this patient shortened the operation time, effectively reduced bleeding, and did not have blood transfusions, the incidence of postoperative complications would be only about 25%. The advantage of MVR lies in the complete resection of the chamber, which not only improves the local control but also reduces the prolonged operation time and increased blood loss in some patients due to the precise dissection of organs (such as renal vessels) [18]. Thus, contrary to previous understanding, an aggressive surgical strategy may also lead to better short-term prognostic outcomes in selected elderly patients.

Although estimated blood loss and packed RBC transfusion were not independent risk factors for postoperative complications in multivariate analysis, they were also included in this model. Intraoperative blood loss can lead to physiologic fluid shifts, coagulopathy, antibiotic dilution, and the need for transfusions [19]. Also, the detection and management of bleeding points will increase the operative time. Therefore, in addition to adequate preoperative preparations, a visit to an experienced high-volume sarcoma center is especially crucial as a relatively rare disease. As for packed RBC transfusion, our previous reports on the safety of surgery for retroperitoneal solitary fibroids also identified risk factors for postoperative complications [20]. We suggest that postoperative immune disturbances induced by blood transfusion may be a mechanism leading to poor prognosis [21].

The present study has the following shortcomings. First, as a retrospective study, selection bias is inevitable. Second, the nomogram prediction model developed in this study, although internally validated well, needs further external verification to confirm its practicability. Third, many recent studies have reported that preoperative nutritional indicators are also influential factors of complications after RPS, such as preoperative albumin, lymphocytes and CRP, but nutritional indicators were not included in this study due to the lack of data.

## Conclusions

In conclusion, this study is the first to establish a predictive model of nomogram using high flow centers to predict postoperative complications in patients with primary RPS, providing a reference for preoperative counseling, selection of patients at high risk for complications, and early intervention.

## Data Availability

The datasets used and analyzed during the current study are available from the corresponding author upon reasonable request.

## Abbreviations

RPS: Retroperitoneal sarcoma
MVR: Multivisceral resection
ASA: The American Society of Anesthesiologists Physical Status
FNCLCC: The Federal National Cancer Center
AIC: Akaike Information Criteria
c-index: Harrell C's Concordance Index
DCA: Decision curve analysis
